# Shorter respiratory event duration is related to prevalence of type 2 diabetes

**DOI:** 10.3389/fendo.2023.1105781

**Published:** 2023-02-16

**Authors:** Junwei Guo, Lu Dai, Jinmei Luo, Rong Huang, Yi Xiao

**Affiliations:** Department of Pulmonary and Critical Care Medicine, Peking Union Medical College Hospital, Chinese Academy of Medical Sciences, Peking Union Medical College, Beijing, China

**Keywords:** obstructive sleep apnea, type 2 diabetes, respiratory event duration, polysomnography, sleep

## Abstract

**Background:**

Obstructive sleep apnea (OSA) is a heterogeneous sleep disorder often comorbid with metabolic diseases, and type 2 diabetes (T2DM) is one of them. Although apnea hypopnea index (AHI) is currently the diagnostic criteria for OSA severity, a controversial relationship between AHI and T2DM has been found. On the other hand, the duration of apnea–hypopnea events has been shown to be a useful metric for predicting mortality. This study aimed to test whether average respiratory event duration was associated with prevalence of T2DM.

**Methods:**

Patients referred to the sleep clinic were recruited in the study. Baseline clinical characteristics and polysomnography parameters including average respiratory event duration were collected. The association of average respiratory event duration with the prevalence of T2DM was evaluated by univariate and multivariate logistic regression analyses.

**Results:**

A total of 260 participants were enrolled, and 92 (35.4%) had T2DM. Univariate analysis revealed that age, body mass index (BMI), total sleep time, sleep efficiency, history of hypertension, and shorter average respiratory event duration were associated with T2DM. In multivariate analysis, only age and BMI remained significant. While average respiratory event duration was insignificant in multivariate analysis, subtype event analysis showed that shorter average apnea duration was both significant in univariate (OR, 0.95; 95% CI, 0.92–0.98) and multivariate analyses (OR, 0.95; 95% CI, 0.91–0.99). Neither average hypopnea duration nor AHI was associated with T2DM. Significant association (OR, 1.19; 95% CI, 1.12–1.25) was observed between shorter average apnea duration and lower respiratory arousal threshold after multivariate adjustment. However, causal mediation analysis revealed no mediating effect of arousal threshold on average apnea duration and T2DM.

**Conclusion:**

The average apnea duration may be a useful metric in the diagnosis of OSA comorbidity. Shorter average apnea duration indicating poor sleep quality and augmented autonomic nervous system responses might be the potential pathological mechanisms leading to T2DM.

## 1 Introduction

Obstructive sleep apnea (OSA) is a highly prevalent sleep-disordered breathing characterized by repeated upper airway collapse with subsequent intermittent hypoxemia, sympathetic activation, and sleep fragmentation. Long-term untreated OSA has been associated with various comorbidities such as cardiovascular disease (CVD) (1), type 2 diabetes (T2DM) (2), and neurocognitive impairment (3). Epidemiological evidence showed that approximately over 936 million adults aged 30–69 years had OSA, and 425 million were estimated to be moderately to severely affected (4). The related economic costs and disease burden are substantial (5). Among these comorbidities, T2DM has shared risk factors with OSA, such as obesity and aging. Several meta-analyses have found positive dose–response association between OSA, characterized by apnea–hypopnea index (AHI), and T2DM (6–8). Despite the significant association of OSA with poorer glycemic control (9), higher risk of micro- and macrovascular complications (10), and worsening of diabetic neuropathy (11), controversies remain on the appropriate metric for OSA and T2DM.

As the recommended metric in the current research and clinical practice, AHI provides limited information without fully considering the impact of hypoxemia. The degree, duration, and frequency of event-related desaturation could all have a great impact on the disease severity, yet none of these are reflected by AHI. Apart from intermittent hypoxemia, sleep fragmentation is another key pathological characteristic that gains fewer researchers’ attention. Several studies have indicated that AHI could neither fully represent the heterogeneity of OSA nor predict the incidence of OSA complications (12). Treatment of OSA based on AHI showed limited benefits for the prevention or reversal of these complications including heart failure and hyperglycemia (13–15). These shortcomings shed light on the importance of new metrics for disease evaluation and intervention.

Methods and metrics aiming at a more comprehensive OSA evaluation have been proposed in recent years, such as hypoxic burden (16), sleep breathing impairment index (17), specific OSA phenotypes identified by cluster analysis (18), and machine learning. These have shown advantages over AHI on the association with CVD. Respiratory event duration is an easily accessible yet less studied trait of OSA. As an indirect measure of OSA severity, it also reflects the extent of intermittent hypoxemia and hypercapnia. Evidence has been found that short respiratory event duration predicted mortality in both men and women in the Sleep Heart Health Study (19). Event-duration-related phenotype has been shown to be heritable (20), and specific genetic loci in Hispanic/Latino Americans have been discovered (21). This inherited property further underscores its diagnostic value for OSA evaluation.

Despite the promising efforts made in association between OSA and CVD, few studies have demonstrated equivalent clinical value for other metabolic diseases. This study aims to evaluate the relationship between respiratory event duration and T2DM in participants referred to the sleep clinic. We hypothesize that compared to AHI and other traditional hypoxemia indexes, individuals with shorter respiratory event duration could be significantly associated with prevalence of T2DM.

## 2 Materials and methods

### 2.1 Patients

Patients referred for suspected OSA at the sleep center and underwent full-night polysomnography (PSG) from November 2020 to October 2022 were enrolled. The inclusion criteria were (1) age > 18 years old, (2) accomplishing the demographic and sleep questionnaires, and (3) willing to participate and sign the informed consent. The exclusion criteria were (1) receiving OSA treatment including continuous positive airway pressure (CPAP) over a year before enrollment; (2) diagnosis of restless legs syndrome, narcolepsy, or rapid-eye-movement sleep behavior disorder; (3) combining severe hepatic or renal insufficiency or pregnancy; (4) total sleep time <4 h; and (5) other type of diabetes. The study protocol was approved by the ethics committees of PUMCH (JS-3573). The whole procedure was conducted in accordance with the Declaration of Helsinki. Written informed consent was obtained from each participant in this study.

### 2.2 Overnight polysomnography and data collection

Overnight PSG was performed for all patients by a standard device (PSG, Embla N7000, Natus Medical Incorporated, Orlando, FL, USA) from 11 p.m. to 6 a.m. Sleep state, respiratory events, and associated desaturation were evaluated by a skilled sleep laboratory technician following the American Academy of Sleep Medicine standard protocols recommendations (22). AHI (none, AHI < 5; mild, 5 ≤ AHI < 15; moderate, 15 ≤ AHI < 30; severe, AHI ≥ 30) was used to assess the severity of OSA. Apneas were defined as a complete or almost complete cessation of airflow (a reduction in airflow by ≥90% of pre-event baseline), lasting ≥10 s, and hypopneas were defined as a reduction in airflow ≥30% of pre-event baseline, lasting ≥10 s, associated with a ≥3% desaturation. Average apnea–hypopnea event duration (>10 s) was extracted from PSG and was separated as average apnea duration and average hypopnea duration for further analysis. All respiratory events were scored by the same certificated technician. Central apneas and hypopneas were not included in the analysis. Other metrics including percent of time spent with SpO_2_ < 90% (T90), oxygen desaturation index (ODI), and lowest pulse oxygen saturation (LSpO_2_) were obtained as the ancillary description of nocturnal hypoxemia.

Other measurements were based on the questionnaires and clinic examinations. Participants’ demographics, alcohol use, and smoking status were obtained using standard questionnaires. A history of the following medical conditions was collected: diabetes mellitus, chronic obstructive pulmonary disease, and hypertension. Body mass index (BMI) was defined as weight (in kg)/height^2^ (in m^2^). Obesity was defined as BMI ≥ 28 kg/m^2^ (23). Waist-to-hip ratio (WHR) was calculated as waist circumference/hip circumference. CVD was defined as any prevalent heart attack, angina, heart failure, coronary heart disease, transient ischemic attack, stroke, aortic aneurysm, or peripheral vascular disease including claudication. Self-reported questionnaires such as the Pittsburg Sleep Quality Index (PSQI) (24) and Epworth Sleepiness Scale (ESS) (25) were gathered to assess the individuals’ sleep quality and daytime sleepiness. Poor sleep quality was defined as a PSQI score ≥5, while daytime sleepiness was defined as an ESS score ≥ 10. Participants with low arousal threshold (ArTH) were classified by a clinical score, which was validated against the epiglottic catheter method. Participants with at least two of the following conditions were considered to have a possible low ArTH: (1) >58.3% of all respiratory events are hypopneas, (2) >82.5% oxygen saturation (SpO2) nadir, and (3) mild to moderate OSA (26).

### 2.3 Diabetes status

Diabetes status and medication history were gathered by standard questionnaires. Participants with self-reported diabetes, and/or use of medications to treat diabetes, and/or fasting blood glucose ≥ 7.0 mmol/L in the medical records were considered to have diabetes.

### 2.4 Statistical analysis

Baseline characteristics and sleep measurements were summarized by mean ( ± SD) or median (interquartile range, 25%–75%) for continuous variables depending on the data distribution. Categorical variables were presented as frequencies with proportions. Event duration was categorized into quartiles, with the first quartile (Q1: longest event durations) serving as the reference category. Q2–Q4 were arranged by the decreasing order. Baseline differences in continuous demographics and sleep characteristics among quartiles of event duration were assessed by one-way ANOVA when variances were equal and by Wilcoxon/Kruskal–Wallis test when variances were unequal. Differences among categorical variables were assessed by χ^2^ test.

Logistic regression analysis was used to evaluate the association between average respiratory event duration and T2DM. Covariates with p-values < 0.2 after the univariate analysis will be reexamined by the multivariate analysis. Specifically, average apnea–hypopnea event duration was divided into average apnea duration and average hypopnea duration. The associations of these event subtypes with T2DM were also evaluated. Significant continuous variables from the multivariate analysis were converted into categorized (quartiles) variables and re-evaluated in logistic regression analysis. Test for trend was based on variable containing the median value for each quartile.

To further delineate the association of T2DM with AHI, average apnea–hypopnea duration, average apnea duration, and average hypopnea duration, restricted cubic spline (RCS) curves were used to detect the possible non-linear dependence of the relationships. Covariates based on univariate logistic regression analysis were adjusted in RCS analysis. To balance best fit and overfitting of the association, the number of knots, between three and five, was chosen based on the lowest value of the Akaike information criterion (AIC) and Bayesian information criterion (BIC). The location of knots was chosen based on the recommendations from a previous work (27). The Wald chi-square test was used to test the overall association and non-linear association of continuous variables with T2DM.

To explore potential pathological mechanisms underlying the relationship between average apnea duration and T2DM, causal mediation analysis (SAS PROC CAUSALMED) was used to determine the mediation effect of respiratory ArTH. Confidence intervals (CIs) were computed based on the 1,000 bootstrap method.

All statistical tests were conducted in SAS 9.4 (SAS Institute, Inc., Cary, NC, USA) and R (RStudio version 4.2.2). RCS analysis was achieved using a SAS macro developed by Desquilbet et al. (28). A two-sided p-value of <0.05 was considered significant.

## 3 Results

### 3.1 Baseline characters of recruited participants

**Figure 1:**
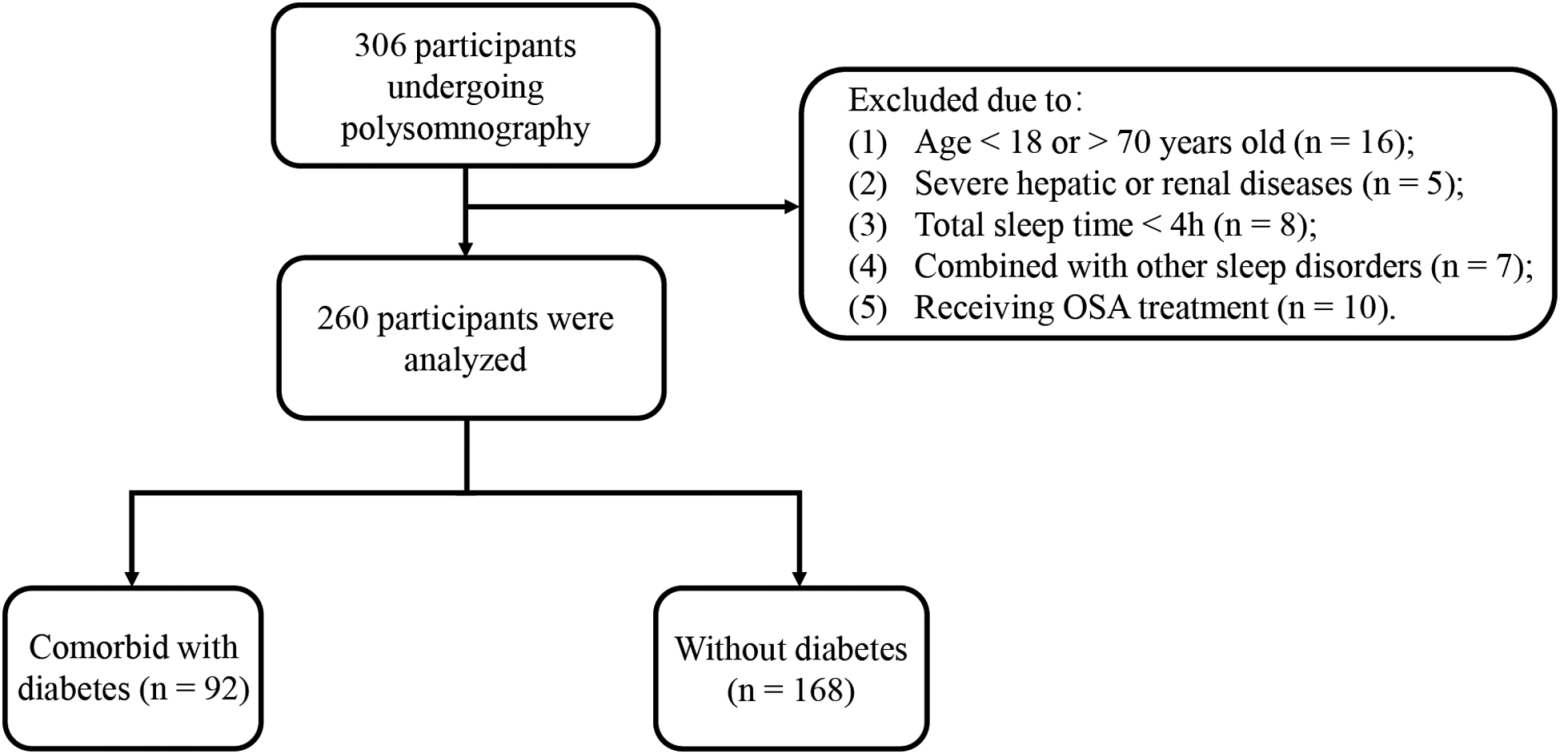
Flowchart of the study population.

The study evaluated 306 participants. A total of 260 people who met the inclusion criteria were recruited. A flow chart for the study population is presented in **Figure 1**. Among the participants, 205 were diagnosed with OSA (78.9%). Men were 79.4%, with a mean age of 44 years old and a median BMI of 27.5 (24.5–30.6) kg/m^2^. A total of 92 (35.4%) participants were confirmed to have comorbid T2DM. **Table 1** and **Supplementary Table 1** display the baseline characteristics of patients by quartile ranges of event duration and by diabetes status. Participants with the shortest average event duration (Q4, range, 12.62–18.80 s) tended to be women with a lower proportion of alcohol use when compared to patients with the longest average event duration (Q1, range, 27.73–59.90 s). Shorter average event duration was also correlated with a smaller proportion of OSA, better hypoxemia metrics (lower ODI, higher LSpO_2_, and shorter T90), lower sleep efficiency, and a higher proportion of patients with lower ArTH. Compared to non-diabetic patients, patients with T2DM were older and had a higher proportion of hypertension history, higher baseline BMI, shorter average apnea duration, and shorter respiratory event duration. Of note, AHI and other hypoxemia metrics, including ODI, LSpO_2_, and T90, were not significantly different between the two groups.

**Table 1:** Baseline characteristics by quartile of average respiratory event duration.

Variables	Q1	Q2	Q3	Q4	p-value
N	65	65	65	65	NA
Range	(27.73, 59.90]	(22.87, 27.73]	(18.80, 22.87]	[12.62, 18.80]	NA
Mean ± SD	33.70 ± 5.93	25.25 ± 1.47	21.15 ± 1.19	16.04 ± 1.70	NA
Age, y	47.03 ± 10.20	48.12 ± 12.53	48.52 ± 12.67	50.51 ± 13.02	0.428
Sex, male, n (%)	60 (92.31)	56 (86.15)	50 (76.92)	44 (67.69)	0.002
Race, Han, n (%)	62 (95.38)	57 (87.69)	62 (95.38)	61 (93.85)	0.326
WHR, male, %	0.96 (0.92-1.00)	0.96 (0.93-0.99)	0.96 (0.92-1.00)	0.97 (0.95-1.00)	0.517
WHR, female, %	0.95 (0.84-0.95)	0.94 (0.88-0.97)	0.94 (0.91-0.97)	0.91 (0.86-0.95)	0.504
BMI, kg/m^2^	28.71 (25.72-31.65)	28.37 (27.14-30.44)	28.41 (26.49-32.85)	28.71 (26.20-31.88)	0.950
BMI ≥ 28, n (%)	38 (58.46)	36 (55.38)	40 (61.54)	38 (58.46)	0.917
Hypertension, n (%)	32 (49.23)	21 (32.31)	37 (56.92)	32 (49.23)	0.037
CVD, n (%)	12 (18.46)	10 (15.38)	20 (30.77)	16 (24.62)	0.155
Tobacco smoke					0.222
Never, n (%)	33 (50.77)	38 (58.46)	37 (56.92)	48 (73.85)	
Former, n (%)	15 (23.08)	12 (18.46)	11 (16.92)	7 (10.77)	
Current, n (%)	17 (26.15)	15 (23.08)	17 (26.15)	10 (15.38)	
Alcohol use					0.026
Never, n (%)	11 (16.92)	18 (27.69)	16 (24.62)	22 (33.85)	
Sometimes, n (%)	43 (66.15)	35 (53.85)	41 (63.08)	37 (56.92)	
Often, n (%)	11 (16.92)	12 (18.46)	8 (12.31)	6 (9.23)	
ESS > 10, n (%)	35 (53.85)	38 (58.46)	34 (52.31)	35 (53.85)	0.824
PSQI ≥ 5, n (%)	55 (84.62)	56 (86.15)	53 (81.54)	59 (90.77)	0.501
Total sleep time, min	407.50 (363.20-447.10)	407.00 (358.00-436.20)	405.50 (363.00-439.50)	384.20 (351.00-416.00)	0.197
Sleep efficiency	92.0 (81.1-96.1)	90.1 (82.0-94.7)	90.4 (82.1-94.8)	86.7 (76.5-91.6)	0.027
AHI,/h	54.60 (26.60-67.90)	31.40 (12.80-56.70)	18.60 (5.00-36.80)	6.40 (2.80-12.50)	<0.001
OSA, n (%)	62 (95.38)	58 (89.23)	49 (75.38)	36 (55.38)	<0.001
ODI,/h	49.60 (20.80-64.80)	23.60 (10.00-46.10)	13.40 (4.50-30.30)	4.30 (1.30-11.90)	<0.001
LSpO_2_, %	75 (69-84)	84 (77-89)	88 (82-91)	91 (88-93)	<0.001
T90, %	3.0 (0.5-8.6)	1.1 (0-4.9)	0 (0-0.6)	0 (0-0)	<0.001
Avg. AP Dur, s	32.20 (28.40-36.00)	23.30 (20.80-25.20)	19.00 (16.00-21.30)	14.60 (13.30-16.00)	<0.001
Avg. HP Dur, s	29.00 (23.10-32.90)	27.30 (25.20-32.00)	22.95 (21.50-24.40)	17.70 (16.10-19.50)	<0.001
Low ArTH, n (%)	15 (23.08)	28 (43.08)	35 (53.85)	57 (87.69)	<0.001

WHR, waist hip ratio; BMI, body mass index; CVD, cardiovascular disease; ESS, Epworth Sleepiness Scale; PSQI, Pittsburg Sleep Quality Index; AHI, apnea hypopnea index; ODI, oxygen desaturation index; LSpO_2_, lowest pulse oxygen saturation; T90, time spent with SpO_2_ < 90%; Avg. AP Dur, average apnea duration; Avg. HP Dur, average hypopnea duration; ArTH, arousal threshold.

Differences among quartiles were assessed by one-way ANOVA when variances were equal. Differences among quartiles were assessed by Wilcoxon/Kruskal–Wallis test when variances were unequal. Differences among quartiles in proportions were assessed by χ^2^ test. NA, No analysis; OSA, obstructive sleep apnea.

### 3.2 Association between event duration and T2DM

We performed univariate and multivariate logistic regression analyses to evaluate the association of average respiratory event duration and AHI with T2DM. Demographic characteristics, sleep indicators, and respiratory event duration were examined. The results are presented in **Table 2**. In the univariate analysis, participants’ age, BMI, total sleep time, and sleep efficiency were associated with T2DM. Average respiratory duration was also correlated with T2DM, with odds ratios (ORs) of 0.95 (95% CI, 0.92–0.99), which suggested that decreasing the average respiratory duration per second increased the odds of T2DM by approximately 5%. Significant covariates, together with gender, were considered in the multivariate analysis. Only age (OR, 1.06; 95% CI, 1.03–1.09) and BMI (OR, 1.11; 95% CI, 1.05–1.08) remained significant. Average respiratory duration no longer remained in the final model. In contrast, traditional metrics like AHI, ODI, LSpO_2_, and T90 were not significant in the univariate analysis (**Table 2**).

**Table 2:** Logistic regression analysis of average respiratory event duration and AHI with prevalence of diabetes.

Variables	Univariate analysis OR (95% CI)	p	Multivariate analysis for Avg. Event Dur OR (95% CI)	p	Multivariate analysis for Avg. AP Dur OR (95% CI)	p	Multivariate analysis for Avg. AP Dur quartiles OR (95% CI)	p
Age	1.04 (1.02-1.06)	< 0.001	1.06 (1.03-1.08)	< 0.001	1.05 (1.03-1.08)	< 0.001	1.06 (1.03-1.08)	< 0.001
Gender	1.57 (0.84-2.94)	0.158	–	–	–	–	–	–
Race	2.00 (0.64-6.26)	0.234	–	–	–	–	–	–
BMI	1.08 (1.03-1.14)	0.002	1.12 (1.06-1.18)	< 0.001	1.12 (1.06-1.18)	< 0.001	1.12 (1.06-1.18)	< 0.001
WHR	1.09 (0.02-52.09)	0.966	–	–	–	–	–	–
Alcohol use	1.13 (0.55-2.32)	0.736	–	–	–	–	–	–
Current smoking	1.11 (0.61-2.03)	0.728	–	–	–	–	–	–
History of CVD	1.39 (0.77-2.53)	0.280	–	–	–	–	–	–
Hypertension	1.82 (1.09-3.04)	0.022	–	–	–	–	–	–
Total sleep time	0.99 (0.99-1.00)	0.079	–	–	–	–	–	–
Sleep efficiency	0.98 (0.96-1.00)	0.055	–	–	–	–	–	–
Avg. Event Dur	0.95 (0.92-0.99)	0.015	–	–	–	–	–	–
Avg. AP Dur	0.95 (0.92-0.98)	0.005	–	–	0.95 (0.92-0.99)	0.016	–	–
Avg. HP Dur	0.99 (0.95-1.02)	0.418	–	–	–	–	–	–
Avg. AP Dur quartiles			–	–	–	–	–	–
Q1 (Ref, longest)	1	–	–	–	–	–	1	–
Q2	0.78 (0.36-1.73)	0.5459	–	–	–	–	0.64 (0.28-1.49)	0.303
Q3	1.92 (0.93-3.99)	0.0791	–	–	–	–	1.53 (0.71-3.30)	0.283
Q4	2.45 (1.18-5.10)	0.0163	–	–	–	–	2.22 (1.02-4.81)	0.044
P for trend	0.006						0.031	
LSpO_2_	1.01 (0.99-1.04)	0.417	–	–	–	–	–	–
ODI	1.00 (0.99-1.01)	0.839	–	–	–	–	–	–
T90	0.98 (0.96-1.01)	0.243	–	–	–	–	–	–
AHI	1.00 (0.99-1.01)	0.960	–	–	–	–	–	–

BMI, body mass index; WHR, waist-to-hip ratio; CVD, cardiovascular disease; Avg. Event Dur, average respiratory event duration; Avg. AP Dur, average apnea duration; Avg. HP Dur, average hypopnea duration; LSpO_2_, lowest pulse oxygen saturation; ODI, oxygen desaturation index; T90, time spent with SpO_2_ < 90%; AHI, apnea hypopnea index; OR, odds ratio; CI, confidence interval.

The multivariate analysis included covariates with p-values < 0.2 in the univariate analysis (age, gender, BMI, total sleep time, sleep efficiency, and hypertension).

The average apnea–hypopnea event duration was then divided into average apnea duration and average hypopnea duration. These two metrics were added to the logistic regression analysis. The results are listed in **Table 2**. While no association was found between average hypopnea duration and T2DM, a significant association was observed in both univariate (OR, 0.95; 95% CI, 0.92–0.98) and multivariate analysis (OR, 0.95; 95% CI, 0.92–0.99) for average apnea duration. The average apnea event duration was further transformed to a categorized (quartiles) variable, with the first quartile (Q1: longest apnea duration) serving as the reference category (**Table 2**). Compared to the reference group (Q1), participants with the shortest average apnea duration (Q4) had a significant association with T2DM in univariate analysis (OR, 2.45; 95% CI, 1.18–5.10). The significance remained in multivariate analysis (OR, 2.22; 95% CI, 1.02–4.81), which suggested that participants with an average apnea duration of <15.80 s (Q4) had 2.2-fold higher odds of T2DM than those with an average apnea duration of more than 25.75 s (Q1).

### 3.3 Restricted cubic spline analysis

**Figure 2:**
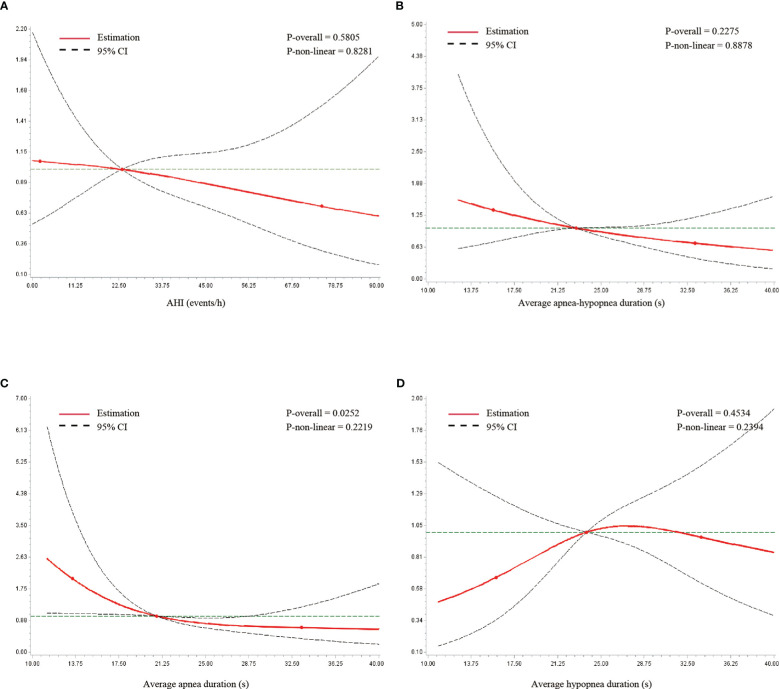
Restricted cubic spline analysis of metrics with type 2 diabetes. **(A)** AHI, **(B)** average apnea–hypopnea duration, **(C)** average apnea duration, and **(D)** average hypopnea duration. The overall and non-linear association were estimated by Wald chi-square test.

The association of T2DM with average event duration and AHI was further evaluated by RCS analysis. Based on AIC and BIC criteria, three knots were chosen for the analysis. The association between average apnea duration on a continuous scale and T2DM prevalence was nearly linear. A shorter duration was associated with a higher prevalence of T2DM (**Figure 2C**). The Wald chi-square test for overall association was significant (p = 0.025). Similar linear associations were observed in AHI and average apnea–hypopnea duration (**Figures 2A, B**). The association between average hypopnea duration and T2DM was U-shaped (**Figure 2D**). However, none of these three variables were significantly associated with T2DM in the Wald chi-square tests.

### 3.4 Sensitivity analysis

Although respiratory event duration has been proven to be an independent and heritable metric, it may have high variability in those non-OSA participants with less respiratory events. We performed a logistic analysis restricted to OSA patients (n = 205). The multivariate analysis remained significant for average apnea duration (OR, 0.94; 95% CI, 0.90–0.99, p = 0.010).

### 3.5 Relationship of average apnea duration with BMI and WHR

Abdominal obesity could affect both OSA patterns and diabetes incidence. We used BMI and WHR as substitutional metrics for abdominal obesity. Pearson correlation analysis was used to evaluate the association between average apnea duration and BMI and WHR in men and women separately (**Supplementary Figure 1**). No significant correlation was found neither in the male (r = −0.117, p = 0.092 for BMI and r = −0.011, p = 0.874 for WHR) nor in the female subgroup (r = −0.080, p = 0.579 for BMI and r = −0.064, p = 0.661 for WHR).

### 3.6 Association between average apnea duration and lower arousal threshold

Shorter event duration may be associated with lower ArTH, which could be a contributing factor to T2DM. Baseline characteristics across quartiles showed that average event duration and ArTH were both affected by anthropometric data. Logistic regression was applied to evaluate the adjusted association between average apnea duration and ArTH. After adjusting for potential covariates including age, gender, and BMI, average apnea event duration was significantly associated with ArTH (OR, 1.19; 95% CI, 1.12–1.25), which implied that participants with shorter average apnea duration tended to have lower ArTH. However, in causal mediation analysis, the result showed that no indirect effect of lower ArTH on diabetes was observed (OR of natural indirect effect, 1.002; p= 0.852; **Supplementary Table S2)**.

## 4 Discussion

This cross-sectional study was designed to evaluate whether average respiratory event duration, compared to AHI, could be better associated with T2DM and serve as a potential marker for OSA clinical outcomes. The major findings are listed as follows. First, shorter average apnea–hypopnea duration and average apnea duration were both significantly associated with T2DM in univariate analysis. Particularly, in multivariate analysis, the association of T2DM with shorter average apnea duration, age, and BMI remained significant. No significance was observed regarding the association between average hypopnea duration and T2DM. Second, traditional indexes such as AHI, ODI, and T90 were not associated with T2DM. Third, shorter average apnea duration was associated with poor sleep quality and lower arousal threshold, which might be the potential mechanism for T2DM, but no mediation effect was observed for lower ArTH. The mechanism needed to be confirmed by more evidence.

Sleep-related disorders and T2DM are highly prevalent, especially in the elderly. There is certain evidence from experiments and clinical research that OSA is associated with T2DM (29, 30). As the main pathological traits of OSA, intermittent hypoxemia and sleep fragmentation are also considered the pathways to diabetes in OSA patients (2). Individuals with diabetes, on the other hand, could have worse OSA by adversely affecting the central control of respiration or upper airway neural reflexes that promote airway patency (31, 32). Despite this bilateral relationship and many shared risk factors, controversy still exists over the proper metrics for clinical practice in disease prediction and treatment evaluation. Both diseases are heterogeneous, with different indexes, stages, and subtypes, resulting in inconsistencies in the correlation. Some studies, but not all, found a dose–response association between OSA severity characterized by AHI and T2DM. Other studies claimed that only AHI during REM stage was independently associated with insulin resistance and levels of HbA1c (33–35). A cohort study carried out by Appleton et al. (36) examined the relationship between indices of undiagnosed OSA and the development of abnormal glycemic control. The results suggested that mean SpO_2_, rather than AHI or T90, was associated with abnormal glycemic metabolism. Another study reported that T90, instead of nadir SpO_2_, was a risk factor for T2DM (37). Besides these traditional metrics, Ding et al. (38) identified certain phenotypes of OSA based on cluster analysis using PSG data. In comparison to AHI, PSG phenotypes improved T2DM risk prediction. These inconsistencies make it necessary to find better parameters for OSA evaluation.

Apart from intermittent hypoxemia, sleep fragmentation is another key pathological mechanism of OSA. Some studies demonstrated a significant relationship between sleep fragmentation and glucose metabolism (39), but some found negative results (40), especially when the arousal index was used to assess sleep fragmentation (41). New sleep fragmentation metrics, such as the odds ratio product (42), arousal intensity (43), and oxygen desaturation rate (44), have been proposed in recent years. Some of them have been linked to poor cardiovascular outcomes. Nevertheless, none of these studies reported an association with T2DM.

As an indirect reflection of sleep fragmentation and intermittent hypoxemia, in contrast to the metrics mentioned above, respiratory event duration is readily accessible, yet less studied. In theory, a shorter event duration is associated with lighter sleep depth, lower arousal threshold, or higher loop gain. All of these changes can lead to sleep fragmentation and excess sympathetic tone, which are closely connected to daytime sleepiness, hypertension, and many other adverse clinical outcomes. In addition, this index has been proven to be associated with certain genetic loci found in Hispanic/Latino Americans (21). Butler et al. (19) found shorter event duration to be a risk factor for mortality in men and women after multivariate adjustment. A shorter event duration could be a heritable phenotype that is valuable for disease identification. The assumption needs more evidence for validation. In the study carried out by Ding et al. (38) mentioned above, patients with “PLMS” phenotype, which was another marker of sympathetic activation, independently predict risk of T2DM. However, they were unable to find similar result in patients with “arousal and poor sleep” phenotype. Our cross-sectional study found that shorter average apnea duration was a significant risk factor for T2DM. The result remained significant in multivariate analysis. The RCS analysis showed this almost linear relationship, further proving its prognostic value for T2DM.

Unlike the result found in the work of Butler et al. (19), the subtype of average respiratory event duration analysis showed significance only in average apnea duration after multivariate adjustment. The main differences between apnea and hypopnea are the degree of upper airway obstruction and related oxygen desaturation. In terms of the degree, duration, and time to the lowest SpO_2_, apnea-related desaturation may be more severe than hypopnea-related desaturation. The shorter time to the lowest SpO_2_, known as the faster oxygen desaturation rate, has been proven to be a potential indicator for hypertension (44). A cross-sectional study in OSA patients with diabetes also revealed a significant increase in apnea events compared to matched patients without diabetes, suggesting a different pattern of sleep breathing in T2DM patients (45). Some studies also focused on the clinical differences between apnea- and hypopnea-dominant OSA patients. Apnea-predominant patients had more severe OSA disease (46) and slightly higher PAP levels (47) than hypopnea-predominant patients. The more intensive desaturation resulting from apnea rather than hypopnea may play a critical role in insulin resistance and hyperglycemia.

Besides these two mechanisms, abdominal obesity has been proposed as a potential pathway towards CVD and other clinically adverse outcomes in OSA, which was well illustrated by Jun (48). Patients with abdominal obesity tend to have a lower functional residual capacity, which leads to a reduction in baseline SpO_2_ and predisposes to deeper and faster desaturations during respiratory events. Compared to BMI-matched persons with greater subcutaneous fat mass, patients with abdominal obesity have increased visceral adipose tissue (VAT), in which adipocytes are more active and secrete more pro-inflammatory cytokines. Increased VAT also leads to upper airway obstruction because of fat accumulation in the soft tissue. However, no significant correlation of average apnea duration with BMI or WHR was found in this study. The relationship of VAT mass with BMI and WHR varies with age, sex, and ethics. Using BMI or WHR as substitutional metrics may not fully represent patients with abdominal obesity (49).

Baseline characteristics showed that participants with shorter average event durations were associated with lower sleep efficiency and less total sleep time. Short event duration may represent an instability of ArTH. OSA patients with comorbid ischemic stroke may benefit from drugs aiming at low ArTH (50). We further distinguished patients with a lower respiratory ArTH based on PSG information validated by Edwards et al. (26). After adjusting for age, gender, and BMI, the association between average apnea duration and lower ArTH remained significant in logistic regression models. The result highlighted the close relationship between average event duration and ArTH, which could be the potential mechanism of shorter event duration towards T2DM. However, causal mediation analysis showed no significant indirect effect of lower ArTH on presence of diabetes. Participants with lower ArTH were identified by an indirect method. The actual arousal threshold measured by the epiglottic catheter may still be required. The negative result may also come from the relatively small sample size of this study. The relationship of event duration with the pathological changes in OSA, including loop gain and sleep structures, and the subsequent mechanisms towards T2DM still need to be tested in future research.

The current study has several strengths and limitations. The study evaluated an easily accessible yet less studied metric, average respiratory event duration, and emphasized the relationship of average apnea duration with T2DM. The results remained after adjustments for multiple covariates and were not influenced by OSA status, which proved robustness of our result. Although the measurement errors of this metric due to inter- or intra-rater reliability still need to be addressed, the convenience provided the possibility of wide application for clinical practice. The positive result of average apnea duration sheds light on the complex mechanism of T2DM compared to CVD. The combination of intermittent hypoxemia and sleep fragmentation may be an effective method to seek a better prognostic marker for OSA. However, several limitations exist. First, this study was conducted in a single center with a relatively small sample size and included fewer female participants. Selection bias may exist, and the results need to be further verified. Due to the cross-sectional design, a causal relationship could not be established. Second, diabetes was confirmed by questionnaires or medical records; some undiagnosed diabetes may be missed without serum blood tests. Another limitation was the accuracy of event duration measured in participants with less respiratory events. The night-to-night variability of event duration in non-OSA participants might be substantial. Repeated and longitudinal sleep monitoring in larger sample including non-OSA patients is needed. Lastly, the mechanism behind average event duration is still unclear. Although the result remained significant in non-OSA participants, the minor influence of less respiratory events could not be totally explained by intermittent hypoxemia and sleep fragmentation. More work should be done on the pathological mechanisms of sympathetic activation and oxidative stress. The interaction of factors such as obesity, desaturation rate, and degrees of airflow limitation with event duration is also needed in the future study.

## 5 Conclusion

This study shows that average respiratory event duration, instead of AHI, is significantly associated with T2DM. Participants with shorter average apnea duration have a close relationship with T2DM independent of multiple confounders. Shorter average event duration may represent a certain phenotype of patients characterized by intermittent hypoxemia and poor sleep quality. Together with previous studies, average event duration could be a valuable marker for the prediction of OSA complications different from traditional indices. More research is needed to explore the pathological mechanism behind event duration and its relevance to diverse metabolic diseases.

## Data availability statement

The raw data supporting the conclusions of this article will be made available by the authors, without undue reservation.

## Ethics statement

The studies involving human participants were reviewed and approved by Ethics committees of Peking Union Medical College Hospital. The patients/participants provided their written informed consent to participate in this study.

## Author contributions

JG performed the research, collected the data, and wrote the paper. LD assisted in collecting the data. JL and RH contributed to data analysis. YX contributed to supervision of this study and revision of the manuscript. All authors contributed to the article and approved the submitted version.

## Supplementary material

Supplementary Figure 1 Associations average respiratory duration and with BMI and WHR. **(A)** BMI in male, **(B)** WHR in male, **(C)** BMI in female, **(D)** WHR in female. Correlation was estimated by Pearson coefficient. BMI, body mass index; WHR, waist-to-hip ratio.
