# Supplementary figures and images for: Shorter respiratory event duration is related to prevalence of type 2 diabetes
Source: Front Endocrinol (Lausanne). 2023 Feb 16;14:1105781. doi: 10.3389/fendo.2023.1105781 (PMC9978406; doi:10.3389/fendo.2023.1105781)

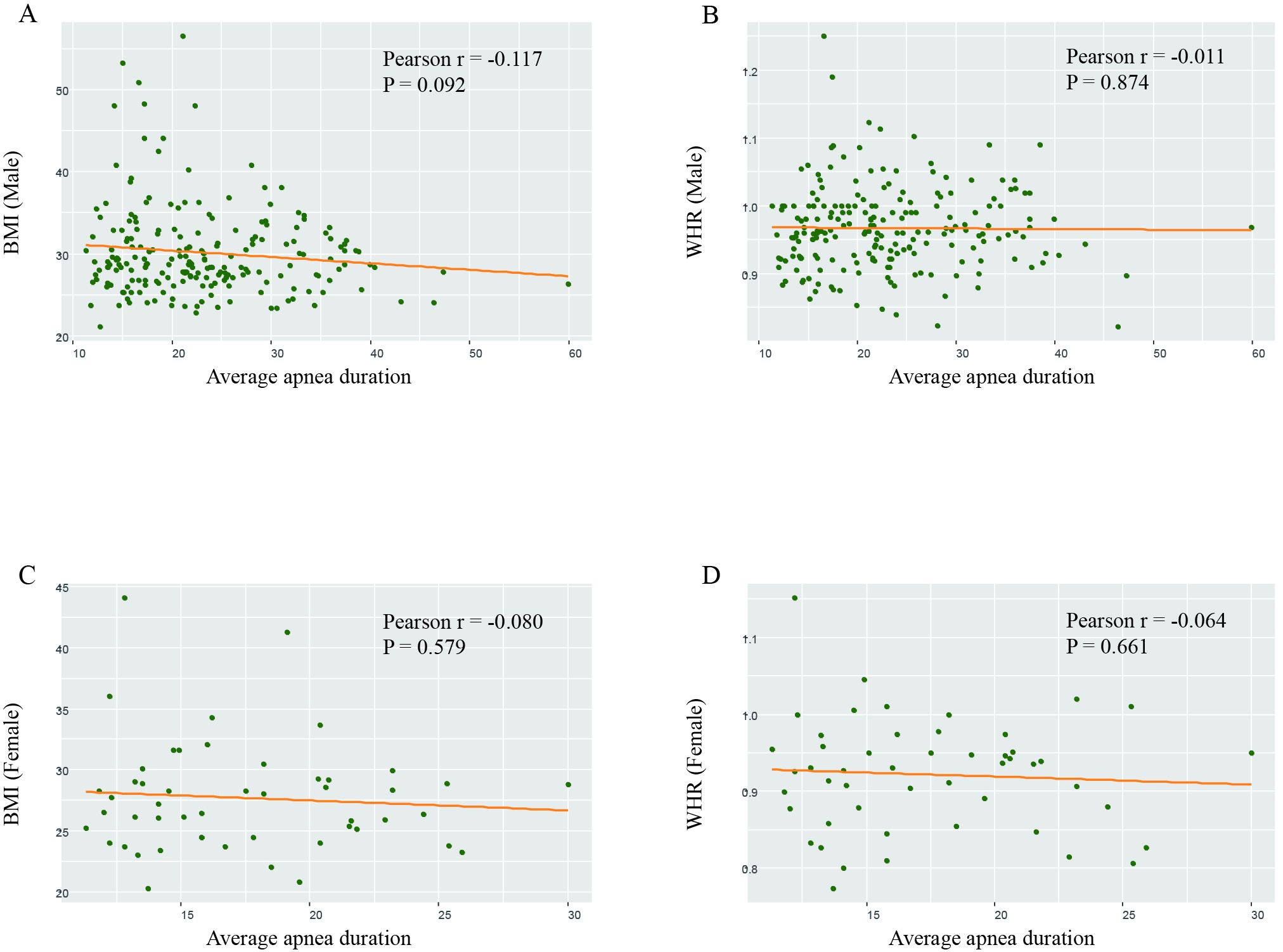

Supplement: Supplementary Figure 1 — Associations average respiratory duration and with BMI and WHR. (A) BMI in male, (B) WHR in male, (C) BMI in female, (D) WHR in female. Correlation was estimated by Pearson coefficient. BMI, body mass index; WHR, waist-to-hip ratio. [file Image_1.jpeg]
