# Supplementary material for: Shorter respiratory event duration is related to prevalence of type 2 diabetes
Source: Front Endocrinol (Lausanne). 2023 Feb 16;14:1105781. doi: 10.3389/fendo.2023.1105781 (PMC9978406; doi:10.3389/fendo.2023.1105781)
Supplement: Supplementary file 2 [file Table_1.docx]

TABLE S1 Baseline demographic and sleep parameters stratified by diabetes status

| Variables | Overall (n = 260) | Diabetes (n = 92) | No Diabetes (n = 168) | P value |
| --- | --- | --- | --- | --- |
| Age, y | 48.55 ± 12.15 | 52.24 ± 11.16 | 46.52 ± 12.23 | <0.001 |
| Sex, male, n (%) | 210 (80.77) | 70 (76.09) | 140 (83.33) | 0.156 |
| Race, Han, n (%) | 242 (93.08) | 88 (95.65) | 154 (91.67) | 0.310 |
| WHR, male, % | 0.97 ± 0.06 | 0.97 ± 0.08 | 0.97 ± 0.05 | 0.556 |
| WHR, female, % | 0.92 ± 0.07 | 0.92 ± 0.06 | 0.92 ± 0.08 | 0.809 |
| BMI, kg/m^2^ | 28.49 (26.36-31.62) | 29.86 (26.20-34.45) | 28.36 (26.39-30.44) | 0.013 |
| BMI ≥ 28, n (%) | 152 (58.46) | 59 (64.13) | 93 (55.36) | 0.170 |
| Hypertension, n (%) | 122 (46.92) | 52 (56.52) | 70 (41.67) | 0.022 |
| CVD, n (%) | 58 (22.31) | 24 (26.09) | 34 (20.24) | 0.279 |
| Tobacco Smoke |  |  |  | 0.240 |
| Never, n (%) | 156 (60) | 59 (64.13) | 97 (57.74) |  |
| Past, n (%) | 45 (17.31) | 11 (11.96) | 34 (20.24) |  |
| Current, n (%) | 59 (22.69) | 22 (23.91) | 37 (22.02) |  |
| Alcohol use |  |  |  | 0.357 |
| Never, n (%) | 67 (25.77) | 28 (30.43) | 39 (23.21) |  |
| Sometimes, n (%) | 156 (60) | 50 (54.35) | 106 (63.10) |  |
| Often, n (%) | 37 (14.23) | 14 (15.22) | 23 (13.69) |  |
| TST, min | 403.20 (355.75-436.70) | 397.25 (349.25-430.50) | 404.30 (362.35-440.80) | 0.125 |
| Sleep efficiency, % |  |  |  |  |
| ESS > 10, n (%) | 142 (54.62) | 56 (60.87) | 86 (51.19) | 0.134 |
| PSQI ≥ 5, n (%) | 223 (85.77) | 82 (89.13) | 141 (83.93) | 0.251 |
| AHI, /h | 23.35 (5.95-54.95) | 22.05 (6.60-52.75) | 24.00 (5.35-57.50) | 0.877 |
| AHI ≥ 5, n (%) | 205 (78.85) | 77 (83.70) | 128 (76.19) | 0.157 |
| ODI, /h | 16.05 (4.55-46.90) | 16.65 (5.85-45.85) | 15.95 (3.90-48.85) | 0.561 |
| LSpO_2_, % | 86 (77-90) | 86 (78.5-91) | 86 (76-90) | 0.652 |
| T90, % | 0.25 (0-3.50) | 0.25 (0-2.45) | 0.25 (0-4.50) | 0.723 |
| Avg. Event Dur, s | 22.87 (18.82-27.86) | 21.99 (18.38-27.18) | 23.60 (19.47-28.28) | 0.018 |
| Avg. Event Dur quartiles |  |  |  |  |
| Q1 (longest) | 32.13 (29.71-35.91) | 30.73 (29.11-32.33) | 33.12 (30.08-36.87) | 0.055 |
| Q2 | 24.90 (23.86-26.65) | 24.90 (23.67-27.09) | 24.97 (23.86-26.65) | 1.000 |
| Q3 | 21.41 (20.18-22.18) | 20.88 (20.21-22.15) | 21.57 (20.00-22.31) | 0.287 |
| Q4 | 16.23 (15.02-17.28) | 15.42 (14.12-17.83) | 16.58 (15.46-17.28) | 0.126 |
| Avg. AP Dur, s | 20.75 (15.80-25.75) | 18.55 (14.40-23.45) | 21.70 (16.20-27.25) | 0.002 |
| Avg. HP Dur, s | 23.75 (19.20-28.70) | 23.25 (19.50-27.20) | 24.05 (19.20-28.90) | 0.458 |
| Low ArTH, n (%) | 135 (51.92) | 50 (54.35) | 85 (50.60) | 0.563 |

WHR, waist hip ratio; BMI, body mass index; CVD, cardiovascular disease; TST, total sleep time; ESS, Epworth Sleepiness Scale; PSQI, Pittsburg Sleep Quality Index; AHI, apnea hypopnea index; ODI, oxygen desaturation index; LSpO_2_, lowest pulse oxygen saturation; T90, time spent with SpO_2_ < 90%; Avg. Event Dur, average respiratory event duration; Avg. AP Dur, average apnea duration; Avg. HP Dur, average hypopnea duration; ArTH, arousal threshold.
