# Supplementary material for: Shorter respiratory event duration is related to prevalence of type 2 diabetes
Source: Front Endocrinol (Lausanne). 2023 Feb 16;14:1105781. doi: 10.3389/fendo.2023.1105781 (PMC9978406; doi:10.3389/fendo.2023.1105781)
Supplement: Supplementary file 3 [file Table_2.docx]

TABLE S2 Causal mediation analysis of arousal threshold in association between average apnea duration and T2DM.

|  | Estimate | 95% CI  by bootstrap method | P |
| --- | --- | --- | --- |
| Odds ratio total effect | 0.95 | 0.91-0.98 | 0.004 |
| Odds ratio controlled direct effect (CDE) | 0.95 | 0.90-0.99 | 0.007 |
| Odds ratio natural direct effect (NDE) | 0.95 | 0.90-0.99 | 0.007 |
| Odds ratio natural indirect effect (IDE) | 1.00 | 0.99-1.12 | 0.852 |
